# Semaglutide from Bench to Bedside: The Experimental Journey Towards a Transformative Therapy for Diabetes, Obesity and Metabolic Liver Disorders

**DOI:** 10.3390/medsci13040265

**Published:** 2025-11-12

**Authors:** Ralf Weiskirchen, Amedeo Lonardo

**Affiliations:** 1 Institute of Molecular Pathobiochemistry, Experimental Gene Therapy and Clinical Chemistry (IFMPEGKC), RWTH University Hospital Aachen, D-52074 Aachen, Germany; 2 Ospedale Civile di Baggiovara (-2023), Department of Internal Medicine, AOU Modena, 41100 Modena, Italy

**Keywords:** GLP-1 receptor agonist, heart failure, MACE, MASLD/MASH, obesity, semaglutide, type 2 diabetes, translational pharmacology, STEP and SUSTAIN clinical trials, weight management

## Abstract

Background/Objectives: Type 2 diabetes and obesity present escalating global health and economic challenges, highlighting the need for therapies that can effectively manage glycemic levels and reduce excess adiposity. Semaglutide, a glucagon-like peptide-1 receptor (GLP-1R) agonist available in subcutaneous or oral formulation, has quickly evolved from a theoretical concept to a crucial component of modern metabolic care. This review explores the comprehensive development journey of semaglutide, drawing on evidence from medicinal chemistry, animal studies, initial human trials, the pivotal SUSTAIN and STEP programs, and real-world post-marketing surveillance. Methods: We conducted a detailed analysis of preclinical data sets, Phase I–III clinical trials, regulatory documents, and pharmaco-epidemiological studies published between 2008 and 2025. Results: Through strategic molecular modifications, such as specific amino-acid substitutions and the addition of a C18 fatty-diacid side chain to enhance albumin binding, the half-life of the peptide was extended to approximately 160 h, allowing for weekly dosing. Studies in rodents and non-human primates showed that semaglutide effectively lowered blood glucose levels, reduced body weight, and preserved β-cells while maintaining a favorable safety profile. Phase I trials confirmed consistent pharmacokinetics and tolerability, while Phase II trials identified 0.5 mg and 1.0 mg once weekly as the most effective doses. The extensive SUSTAIN program validated significant reductions in HbA1c levels and weight loss compared to other treatments, as well as a 26% decrease in the relative risk of major adverse cardiovascular events (SUSTAIN-6). Subsequent STEP trials expanded the use of semaglutide to chronic weight management, revealing that nearly two-thirds of patients experienced a body weight reduction of at least 15%. Regulatory approvals from the FDA, EMA, and other regulatory agencies were obtained between 2017 and 2021, with ongoing research focusing on metabolic dysfunction-associated steatohepatitis, cardiovascular events, and chronic kidney disease. Conclusions: The trajectory of semaglutide exemplifies how intentional peptide design, iterative translational research, and outcome-driven clinical trial design can lead to groundbreaking therapies for complex metabolic disorders.

## 1. Introduction

### 1.1. The Global Burden of Type 2 Diabetes and Obesity

Type 2 diabetes (T2D) is characterized by chronically elevated glycemic values due to inadequate synthesis and release of insulin from the pancreatic β-cells to meet the demands of the body [1]. T2D is typically associated with risk factors such as a family history of the disease, being overweight, having a sedentary lifestyle, and being over forty-five years old [2]. Currently T2D affects more than 400 million people worldwide, with its future prevalence projected to increase. It is a major cause of blindness, non-traumatic lower limb amputations, chronic kidney disease (CKD), chronic liver disease, certain types of cancers and cardiovascular morbidity, leading to an overall expenditure of $11,500 per patient [3,4]. Driven by persistent hyperglycemia, target organ damage in T2D results from cellular stress, overproduction of reactive oxygen species (ROS), advanced glycation end products (AGEs), and protein kinase C (PKC) activation, ultimately leading to the development of microvascular and macrovascular diseases. These mechanisms collectively trigger and sustain inflammation, atherosclerosis, and organ-specific complications such as retinopathy, nephropathy, neuropathy, metabolic-dysfunction associated steatotic liver disease (MASLD), cardiopathy, and brain disease [5]. T2D is often linked with obesity, hence the term “diabesity” used to describe this common and dangerous combination [6].

Obesity is a chronic-relapsing disease characterized by an excess of adipose tissue resulting from prolonged mismatched energy intake exceeding energy expenditure [7]. Hypercaloric diets and sedentary lifestyles lead to the accumulation of lipids in subcutaneous (sc) fat depots, visceral adipose tissue, liver, muscle, and other ectopic sites [8]. These accumulated lipids release free fatty acids and pro-inflammatory cytokines, leading to systemic, low-grade, subclinical chronic inflammation associated with insulin resistance, exacerbating ectopic fat deposition and metabolic dysfunction, ultimately affecting renal, hepatic, cardiovascular, and endocrine health [7,9,10,11,12]. While not perfect, body mass index (BMI) remains the most commonly used tool for assessing obesity in epidemiological studies [7].

Assessment of the global burden of disease (GBD) 2021 database attributable to high BMI indicates a significant and consistent increase in global rates of obesity in adults over the past 30 years, with major variations among countries and region-specific trends. From 1990 to 2021, high BMI-attributable deaths and Disability Adjusted Life Years (DALYs) have risen significantly for both males and females. Females have seen a substantial increase in the absolute numbers of deaths and DALYs, while males have shown a more significant increase in age-standardized rates of these metrics. Age-standardized rates for women have remained relatively stable, whereas men have experienced a 15.0% increase in death rates and a 31.2% increase in DALYs [13]. Due to various interactions among genetic, economic, psycho-socio-cultural, behavioral, and pharmacological factors, the prevalence of obesity has risen globally, especially among certain racial/ethnic groups and individuals with lower educational attainment and income [14]. Individuals living with obesity face a higher annual financial burden in terms of healthcare expenses and medications compared to those of average weight [15]. The burden of both T2D and obesity has increased at a rapid pace among metabolic disorders [16], highlighting the urgent need to address these two common and closely linked metabolic disorders as a public health priority.

### 1.2. Incretin-Based Therapies: GLP-1 Receptor Agonists in Context

**Figure 1:**
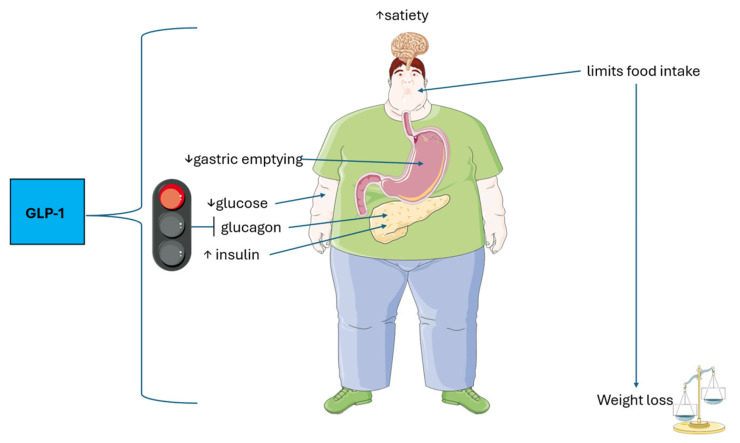
Overview of the main known physiological actions of GLP-1. GLP-1 lowers blood glucose levels by stimulating insulin and inhibiting glucagon secretion. Additionally, it slows gastric emptying, promotes satiety, and reduces appetite, often leading to weight loss. The original illustration was created using Servier Medical ART (SMART) and is licensed under Creative Commons Attribution 4.0 International License (CC BY 4.0).

Glucagon-like peptide 1 (GLP-1) is an incretin hormone synthesized and released by L cells of the small and large bowel post-prandially, particularly after carbohydrate-containing meals [17]. Physiologically GLP-1 stimulates insulin secretion, inhibits glucagon secretion to lower blood glucose levels, slows gastric emptying, increases feelings of satiety, and reduces appetite. This leads to limited food intake and generally results in body weight loss (Figure 1) [18].

Various cell types express GLP-1 receptor (GLP-1R) including pancreatic alpha, beta and delta cells, neurons of the central nervous system (particularly hypothalamus and brain stem), endothelial cells, smooth muscle cells in blood vessels, cardiomyocytes and various regions of the gastrointestinal (GI) tract [19,20]. This diversity of target cells explains why a variety of physiological effects may result from the activity of the GLP-1 hormone, leading to GLP-1R agonists (GLP-1RAs) exerting a range of biological activities.

The discovery of GLP-1 by Joel Habener and Svetlana Mojsov in the 1980s, and Lotte Knudsen’s development sustained-acting versions of this hormone for obesity in 2000, has been summarized by Friedman [21]. GLP-1RAs, by increasing insulin, reducing appetite, delaying stomach emptying, and decreasing liver glucose production are a pharmacological class of drugs (the most prescribed drugs include semaglutide, liraglutide, and dulaglutide) that mimic the natural GLP-1 hormone to help manage T2D and obesity by lowering blood sugar and promoting weight loss [22]. Table 1 summarizes the names, approved indications, route and timing of administration of the GLP-1RAs (Modified from: [23]).

The most common side effects of GLP-1RAs include nausea, vomiting, diarrhea, and constipation, which are often of limited severity and tend to improve over time [24]. Moreover, abdominal pain, anorexia, and headaches may occur and, more rarely, gallstones, pancreatitis, and severe allergic reactions are observed [25].

Pharmacological treatments for obesity provide a valuable adjunct to lifestyle intervention, which often achieves limited weight loss and is difficult to maintain [26]. In this context, GLP-1 RAs are positioned as a viable tool alongside lifestyle changes, often used as a step-up therapy when lifestyle modifications alone are insufficient. They can be used before bariatric surgery to aid in weight loss and improve safety, to complement the results of surgery or to prevent weight regain after surgery [27,28].

### 1.3. Objectives and Scope of the Present Review

Liraglutide played a crucial role in the development of semaglutide, showing the effectiveness of a modified, long-acting GLP-1 analogue for managing diabetes and obesity [29]. Building on the success of liraglutide, semaglutide included a fatty acid moiety to enhance albumin binding and reduce clearance rates. Structural improvements increased albumin affinity, allowing for once-weekly dosing [29,30]. Here, we will comprehensively examine the key steps leading to the discovery and development of semaglutide that was initially developed for T2D, with subsequent recognition of its weight loss benefits [31]. To achieve this, we will incorporate evidence from closely interconnected fields such as medicinal chemistry and animal pharmacology. This will help us chart the human and non-human studies used to characterize cellular targets and pharmacological effects. Additionally, we will explore ongoing investigations into new applications and formulations of semaglutide in humans. Finally, we will address real-world data with a specific emphasis on post-marketing drug monitoring and safety concerns. Table 2 [32,33,34,35,36,37] provides an at-glance chronology of the key pre-clinical discoveries, clinical trials and regulatory decisions that have shaped the journey of semaglutide from its early laboratory design to its current benchmark status in metabolic medicine.

Building on the overview provided in Table 2, the following sections will guide the reader chronologically through the pivotal milestones that have shaped semaglutide’s journey from molecular design to widespread clinical application.

## 2. Molecular Design and Rationale

### 2.1. Native GLP-1: Pharmacological Promise and Limitations

The native incretin hormone GLP-1 is a 30-amino-acid peptide rapidly secreted after meals, capable of stimulating glucose-dependent insulin release. Unfortunately, its pharmacological utility is limited by a plasma half-life of only a few minutes, primarily due to (i) rapid *N*-terminal degradation by the exopeptidase dipeptidyl-peptidase-4 (DPP-4) and (ii) fast renal clearance of the small peptide [32]. The chemical architecture of semaglutide was therefore designed to address these two liabilities while retaining full agonistic activity at the GLP-1 receptor.

### 2.2. Chemical Engineering of Semaglutide

The enzymatic attack by DPP-4 occurs at the Ala8–Glu9 bond of endogenous GLP-1. In semaglutide, alanine at position 8 is substituted with the unnatural residue 2-aminoisobutyric acid (Aib, also termed 2-methyl-alanine). This single change sterically hinders DPP-4, rendering the analogue essentially resistant to proteolysis and preserving its biological integrity for many hours after administration [29,32].

Even when DPP-4-resistant, short linear peptides are removed quickly by glomerular filtration. To slow renal elimination, a C18 di-acid fatty chain is covalently attached to Lys26 through a spacer composed of two 8-amino-3,6-dioxaoctanoic acid units and a glutamic acid moiety. This hydrophobic appendage promotes non-covalent, high-affinity binding to circulating albumin, effectively turning the plasma protein into a reversible depot and raising the terminal half-life to roughly one week, compatible with once-weekly (OW) dosing in humans, which enhances accessibility compared to daily regimens [38].

A second Lys to Arg substitution at position 34 eliminates a potential competing acylation site, ensuring homogeneous conjugation exclusively at Lys26 and simplifying downstream purification. Apart from these deliberate modifications, the remaining residues map precisely onto GLP-1(7-37), preserving the receptor-interacting pharmacophore and maintaining picomolar potency. The final free-acid salt has the empirical formula C_187_H_291_N_45_O_59_ and a molecular weight of 4113 Da, which is still small enough for solid-phase peptide synthesis yet large enough, after acylation, to facilitate binding to serum albumin efficiently [32,37].

### 2.3. Expected Benefits: Extended Half-Life, Once-Weekly Dosing

The three structural interventions (DPP-4 resistance, albumin tethering, single-site acylation) provide several pharmacological advantages:

- Extended exposure: The mean elimination half-life of approximately 160 h allowing for convenient OW sc or once-daily oral regimens.
- Stable receptor stimulation: The prolonged, yet still glucose-dependent insulinotropic action minimizes the risk of hypoglycemia.
- Improved patient adherence: The less frequent injections result in higher compliance compared to daily GLP-1 analogues.
- Manufacturing scalability: The site-specific chemical conjugation after yeast fermentation ensures high product purity at commercial scale.

Therefore, semaglutide represents a rationally engineered peptide whose molecular modifications directly align with the desired clinical profile: sustained glycemic control, significant weight reduction, and OW convenience without compromising safety or effectiveness.

## 3. Preclinical Development

Before semaglutide progressed to first-in-human trials, an extensive non-clinical program was conducted to establish its pharmacodynamic potency, pharmacokinetic behavior, therapeutic efficacy in animal models, and safety margin required by international guidelines. The pivotal studies, conducted under GLP, provided the foundational data that was later included in the investigational new-drug dossiers submitted to the Food and Drug Administration (FDA) and European Medical Agency (EMA).

### 3.1. In Vitro Receptor-Binding and Signaling Assays

Using homogeneous binding competition assays on plasma membranes prepared from BHK cells and BHK cells expressing a reporter construct containing human GLP-1R and the CRE firefly luciferase, it was demonstrated that semaglutide has high affinity for GLP-1R (0.38 ± 0.06 nM) [32]. Human embryonic kidney (HEK-293) cells that over-expressed the GLP-1 receptor were utilized to compare semaglutide with native GLP-1(7-37) [39,40]. Competitive radioligand displacement demonstrated sub-nanomolar affinity, which was comparable to liraglutide and only slightly weaker than the endogenous peptide [32]. In cyclic-AMP accumulation assays, semaglutide displayed full agonism with an EC_50_ in the low-nanomolar range, and β-arrestin recruitment indicated balanced downstream signaling. The incorporation of 2-aminoisobutyric acid at position 8 made the molecule completely resistant to DPP-4 cleavage when incubated with recombinant enzyme for 24 h, while native GLP-1 lost 50% activity within minutes [32,38].

### 3.2. Pharmacokinetics and Metabolic Stability in Animal Models

After sc dosing in rats (0.1–1.0 mg/kg) and cynomolgus monkeys (0.03–0.3 mg/kg), peak plasma concentrations were reached within 8–12 h and modulated food preference, reduced food intake, and caused weight loss without decreasing energy expenditure [41,42,43]. The albumin-binding C18 di-acid sidechain prolonged the elimination half-life to approximately 30 h in rats and 60 h in monkeys, which is roughly 10-fold longer than native GLP-1, while absolute bioavailability exceeded 85% [42]. No sex differences emerged in clearance or steady-state volume of distribution. Metabolite mapping revealed limited biotransformation: the major species in plasma remained intact parent peptide, with minor degradation products formed by endopeptidases rather than DPP-4, corroborating the in vitro stability data [44].

### 3.3. Rodent Efficacy Studies: Glycemic Control and Weight Reduction

The efficacy of semaglutide was first demonstrated in diet-induced obese (DIO) mice [41]. Once-daily administration of semaglutide at a dose of 30 nmol/kg) normalized fed plasma glucose levels within 48 h and reduced HbA1c by approximately 1.5 percentage points over a four-week period. In db/db mice, weekly dosing of semaglutide at a dose of 100 nmol/kg led to a 30–40% decrease in non-fasting glucose levels, preserved pancreatic β-cell area, significantly improved liver damage, and improved gut microbiome compared to the control group that received only the vehicle [45]. Additionally, monitoring of food intake revealed a 15–20% reduction in mice treated with semaglutide, resulting in a cumulative weight loss of 8–10% compared to weight gain in the control group. When compared head-to-head with liraglutide at equivalent molar doses, semaglutide showed superior durability, which was attributed to its extended systemic exposure.

### 3.4. Safety Pharmacology and Toxicology Packages

In order to meet regulatory expectations for first-in-human trials, a thorough safety pharmacology and toxicology program was carried out. This program included genetic toxicology screenings, assessment of organ-system functions, and long-term repeat-dose studies in two different species.

#### 3.4.1. Genotoxicity

Semaglutide tested negative in the bacterial reverse-mutation (Ames) assay, the in vitro mouse-lymphoma assay, and an in vivo rat micronucleus test. This indicates that there is no mutagenic or clastogenic potential at doses up to 5000 µg/plate or 2000 µg/kg body weight [46].

#### 3.4.2. Cardiovascular and Pancreatic Safety

Telemetry studies conducted on conscious dogs showed no effect on heart rate, QTc interval, blood pressure, or arrhythmogenic indices at exposures ≥30-fold higher than anticipated human C_max_. Additionally, dedicated rat and monkey studies, which included detailed histopathology revealed no drug-related myocarditis, valvulopathy, or accelerated atherosclerosis. Pancreatic examinations, utilizing light microscopy and Ki-67 staining, also showed no evidence of ductal proliferation, acinar necrosis, or islet hypertrophy beyond what was observed in age-matched controls [47].

#### 3.4.3. Chronic Dosing Studies

Twenty-six-week repeat-dose studies in rats and 52-week studies in cynomolgus monkeys identified decreased body weight and reduced food intake as the primary pharmacological effects. The no-observed-adverse-effect level (NOAEL) was established at 0.03 mg/kg/week in rats and 0.02 mg/kg/week in monkeys, corresponding to systemic exposures approximately 5- to 8-fold higher than human therapeutic levels. Mild, reversible injection-site reactions were the only consistent local findings. Although the FDA has issued a boxed warning for semaglutide, based on animal studies indicating a risk for thyroid C-cell tumors [48], no target-organ toxicity emerged, and there was no signal for thyroid C-cell hyperplasia, an observation later corroborated in clinical trials.

Collectively, these non-clinical data demonstrated a compelling pharmacological profile and an acceptable safety margin, thereby justifying progression to first-in-human studies and informing the initial dose-escalation schemes.

## 4. First-in-Human (Phase I) Trials

### 4.1. Study Design: Single-Ascending and Multiple-Ascending Doses

Safety and pharmacokinetics of oral semaglutide were investigated by Granhall et al. [33] in two double-blind, randomized, placebo-controlled trials (RCTs). In the single-dose trial, semaglutide exposure was highest when co-formulated with 300 mg of the absorption enhancer sodium N-(8-[2-hydroxybenzoyl] amino) caprylate (SNAC). In the multiple-dose trial, semaglutide exposure showed dose proportionality with 40 mg being twofold higher than 20 mg of oral semaglutide in healthy males, without any differences between healthy males compared to T2D male subjects. Based on this study, oral semaglutide co-formulated with 300 mg SNAC was chosen for further clinical development. Moreover, pharmacokinetics supported once-daily dosing of oral semaglutide.

### 4.2. Pharmacokinetic Profile in Healthy Volunteers vs. Patients with T2D

The pharmacological properties of oral semaglutide were investigated by Xie et al. [49], who found that all doses of oral semaglutide exhibited dose-dependent increases in semaglutide exposure in 32 healthy Chinese adults at steady state. They also noted significantly reduced body weight and fasting plasma glucose levels (*p* = 0.0001 and *p* = 0.0011, respectively) compared to placebo. The most common adverse effects (AEs) reported were GI symptoms. However, there were no severe or confirmed symptomatic hypoglycemic events, serious AEs or deaths reported.

A systematic review of 17 published studies [50] found that semaglutide has a predictable pharmacokinetic profile, with a long half-life supporting OW sc dosing. Both oral and sc forms showed increased area under the curve (AUC) and C_max_ with higher doses. Factors such as food, water volume, and dosing schedules were found to influence drug the absorption of orally administered semaglutide. Drug–drug interactions with semaglutide were limited, requiring no dose adjustment fir subjects with upper GI disorders, impaired renal function, or impaired hepatic function. The study also suggested that exposure to semaglutide may be influenced by body weight, but further investigation is needed to confirm this.

Semaglutide bioavailability showed minor disparities between healthy subjects and those with T2D in terms of clearance and absorption rate, as well as between injection sites [51]. A trial involving 36 healthy Chinese individuals randomly assigned to receive OW sc semaglutide 0.5 mg (*n* = 12), 1.0 mg (*n* = 12), or placebo (*n* = 12) found no dose adjustment was necessary. The primary endpoint of the study was steady-state semaglutide exposure, defined as the area under the curve over a dosing interval at steady state (AUC_0-168 h,SS_) [52].

### 4.3. Tolerability, Adverse Events, and Dose-Limiting Factors

Wharton et al. [53] assessed GI (AEs) occurring after sc 2.4 mg semaglutide OW in adults with overweight or obesity using pooled data from the Semaglutide Treatment Effect in People With Obesity (STEP) 1–3 trials for participants randomized to 68 weeks of semaglutide 2.4 mg (*n* = 2117) or placebo (*n* = 1262). Nausea, diarrhea, vomiting and constipation, the majority of which were non-serious, mild-to-moderate, transient, and occurring more often during or immediately after dose escalation, were more common with semaglutide 2.4 mg than placebo. However, these issues rarely resulted in treatment discontinuation. Mediation analysis showed that semaglutide-induced weight loss was largely independent of GI AEs, and maintenance treatment with semaglutide at the dose of 2.4 mg was well tolerated.

### 4.4. Actions on Gluco-Lipidic Metabolism and Gastric Emptying

The effects of semaglutide on metabolic outcomes and gastric emptying were investigated by Hjerpsted et al. [54] in a double-blind RCT. These authors found that compared to placebo, semaglutide improved fasting and post-prandial glucose levels (as indicated by reduced glucose, insulin, and C-peptide serum concentrations) and lipid metabolism. It also delayed first-hour post meal gastric emptying, which may potentially contribute to a slower entry of glucose into the bloodstream, without affecting overall gastric emptying.

## 5. Proof-of-Concept (Phase II) Programs

### 5.1. Dose–Response Exploration and Optimal Dosing Frequency

The dose–response relationship of semaglutide versus placebo on glycemic control among individuals with T2D was investigated in a 12-week, double-blind phase 2 RCT [30]. A total of 415 individuals were randomly assigned to receive either sc semaglutide OW with or without dose escalation, open-label liraglutide once daily, or placebo. The data showed that after 12 weeks, semaglutide reduced HbA1c in a dose-dependent manner and body weight among individuals with T2D. Mild-to-moderate GI AEs typical of GLP-1 RAs were mitigated by dose escalation. Based on this study semaglutide doses of 0.5 and 1.0 mg OW, with a 4-week dose escalation were selected for phase 3 trials.

O’Neil et al. [55] conducted a double-blind, multicenter phase 2 RCT with 957 non-diabetic adults living with obesity who were randomized to either the active treatment group or a matching placebo. The data showed that all doses of semaglutide induced significant reductions in bodyweight compared to placebo and liraglutide. The treatment was generally well tolerated, with no new safety issues identified. The most commonly observed AE being were dose-related GI symptoms, as expected with GLP-1RAs.

### 5.2. Comparative Efficacy of Liraglutide, Dulaglutide, and Tirzepatide

In their meta-analysis of 16 studies totaling 5997 patients, Karimi et al. [56] compared the effectiveness of semaglutide versus liraglutide, dulaglutide, or tirzepatide. The data indicated that semaglutide reduced HbA1c values compared to liraglutide. However, no significant differences were observed regarding fasting glucose, BMI, and weight change between semaglutide and liraglutide. Semaglutide was more effective than dulaglutide in reducing HbA1c levels and fasting glycemia although no significant differences were found in body weight and BMI changes. When compared to semaglutide tirzepatide was more effective at reducing HbA1c levels without any clear superiority in changes in body weight and fasting blood glucose. Regarding drug transition, moving from liraglutide to semaglutide did not significantly impact HbA1c levels but did induce weight loss and reduce fasting blood glucose. Conversely, transitioning from dulaglutide to semaglutide did not modify HbA1c and weight changes. The study concludes that analyses consistently demonstrate superior efficacy of semaglutide compared to liraglutide in reducing both HbA1c levels and weight. Moreover, it is more effective than dulaglutide in diminishing fasting glycemia. On the other hand, tirzepatide reduces HbA1c values more effectively than semaglutide.

### 5.3. Early Signals in Weight Loss and Cardiometabolic Markers

To investigate changes in body weight and cardiometabolic risk factors after treatment, Wilding et al. conducted an extended analysis of 327 participants from the STEP 1 trial [57]. One year after discontinuing treatment with OW sc 2.4 mg semaglutide in combination with lifestyle intervention, participants regained approximately two-thirds of their initial weight loss, along with similar reversals in cardiometabolic variables. These findings highlight the persistent nature of obesity and suggest that continuous treatment is crucial for maintaining long-term improvements in weight and overall health.

### 5.4. Safety Monitoring: Gastrointestinal Events, Hypoglycemia, Thyroid C-Cell Concerns

A systematic review conducted by Feier et al. [48] analyzed 10 RCTs totaling 14,550 participants (7830 assigned to semaglutide). Additionally, 18 other studies reporting data from the same RCTs were separately discussed and assessed for the incidence of thyroid cancer and the spectrum of semaglutide-associated AE, emphasizing the importance for patient management. The incidence of thyroid cancer among subjects treated with semaglutide indicated no significant risk (<1%). AEs were mostly GI in nature, with nausea (2.05% to 19.95%) and diarrhea (1.4% to 13%) being the most common. Nasopharyngitis and vomiting were also notable, with prevalence rates of 8.23% and 5.97%, respectively. Other AEs included hyperlipasemia (6.5%), headaches (7.92%), anorexia (7%), influenza-like symptoms (5.23%), dyspepsia (5.18%), and constipation (6.91%). Serious AEs ranged from 7% to 25.2%, underscoring the importance of careful monitoring of patients. Importantly, the GI AEs associated with semaglutide are frequent but should not overshadow its treatment benefits, especially considering the low incidence of thyroid cancer.

## 6. Pivotal Phase III Trials: The SUSTAIN Series

The initial Phase 3 program consisted of seven primary SUSTAIN trials (SUSTAIN 1–7), with SUSTAIN 11 being introduced later. SUSTAIN trials 1–5 and 7 evaluated the effectiveness and safety of semaglutide compared to other treatments for type 2 diabetes (T2D), while SUSTAIN 6 specifically examined the cardiovascular safety profile of semaglutide [58].

### 6.1. Trial Architecture and Global Patient Demographics

The SUSTAIN trials investigated the efficacy of semaglutide in over 12,000 adults with T2D through a large-scale, multi-center, double-blind, RCT. Participants had a mean age of 57.4 years (25.8% were aged 65 or above), with 54.2% identifying as male and 78.6% as White [58]. The average duration of T2D was 7.4 years, with a baseline HbA1c of 8.5% and a mean BMI of 35.7 kg/m^2^. The majority presented with hypertension and dyslipidemia and were receiving diabetes polypharmacy. Trial durations were 40 weeks or longer to evaluate long-term outcomes, with participants assigned in a 1:1 ratio to receive OW semaglutide or placebo [58].

### 6.2. Glycemic Endpoints: HbA1c Reduction

To evaluate the effectiveness of semaglutide in achieving glycated HbA1c targets in individuals with T2D while minimizing adverse outcomes such as weight gain, hypoglycemia, and GI side effects, DeVries et al. [59] conducted an analysis of data from phase IIIa SUSTAIN 1 to 5 clinical trials. In total, 3,918 T2D participants were randomized to receive OW sc semaglutide at doses of 0.5 mg or 1.0 mg, or comparators including placebo, sitagliptin 100 mg, exenatide extended release 2.0 mg, or insulin glargine. The proportion of participants achieving HbA1c < 53 mmol/mol (7.0%) without weight gain or severe/BG-confirmed symptomatic hypoglycemia ranged from 47% to 66% with semaglutide 0.5 mg and 57% to 74% with semaglutide 1.0 mg, compared to 7% to 19% for placebo and 16% to 29% for active comparators (all *p* < 0.0001). These findings demonstrate that semaglutide is significantly more effective than comparators in helping patients meet HbA1c targets while avoiding undesirable outcomes.

In head-to-head comparisons, the 1.0 mg dose of semaglutide showed a greater reduction in HbA1c, and body weight compared to the 0.5 mg dose [60].

### 6.3. Weight-Related Outcomes and Adiposity Metrics

In the Semaglutide Effects on Cardiovascular Outcomes in People with Overweight or Obesity (SELECT) trial [61], semaglutide reduced major adverse cardiovascular events by 20% in adults with cardiovascular disease, overweight or obesity, and no diabetes. Over 208 weeks, semaglutide led to sustained weight loss (−10.2%), reduced waist circumference (−7.7 cm), and lower waist-to-height ratio (−6.9%) compared to placebo, with meaningful results across all subgroups. Serious AEs were less frequent with semaglutide, though discontinuation rates increased as baseline BMI decreased. Weight loss and anthropometric improvements persisted for up to four years [61].

The SUSTAIN trials have demonstrated that semaglutide significantly reduced body weight, BMI, and waist circumference compared to placebo. Additionally, this drug led to improvements in cardiometabolic markers, such as blood pressure and cholesterol levels. A substantial proportion of patients treated with semaglutide achieved clinically significant weight loss thresholds (≥5%, ≥10%, ≥15%), with these benefits appearing to be maintained over time, as indicated by data from the SUSTAIN 5 trial [62], which employed a different drug (PHEN/TPM CR). The studies further assessed adiposity indicators, including waist circumference and BMI, revealing that semaglutide contributed to reductions in these metrics and enhancements in health outcomes related to cardiovascular risk and glycemic control.

### 6.4. Cardiovascular Outcome Trial (SUSTAIN-6): Major Adverse CV Events

The SUSTAIN-6 trial [63] showed that OW semaglutide significantly reduced the risk of major adverse cardiovascular events (MACE) by 26% compared to placebo in patients with T2D. This reduction in MACE was consistent across various subgroups based on sex, age, and baseline cardiovascular risk levels. The primary components of MACE (cardiovascular death, nonfatal myocardial infarction, and nonfatal stroke) were also reduced, although the reduction in cardiovascular death alone was not statistically significant in the primary analysis [63].

Building on these findings, Lincoff et al. [35] reported that in their multicenter, double-blind, event-driven superiority RCT of 17,604 individuals (8803 receiving semaglutide and 8801 receiving placebo) with a mean (± SD) duration of exposure of 34.2 ± 13.7 months, weekly sc semaglutide at a dose of 2.4 mg was superior to placebo in reducing the incidence of death from cardiovascular causes, nonfatal myocardial infarction, or nonfatal stroke at a mean follow-up of 39.8 months. (Funded by Novo Nordisk; SELECT ClinicalTrials.gov number NCT03574597).

### 6.5. Safety and Tolerability Overview Across the SUSTAIN and PIONEER Phase IIIa Clinical Trial Programs

Aroda et al. [64] assessed the safety and tolerability of semaglutide in the SUSTAIN (subcutaneous) and PIONEER (oral) phase IIIa trials, which included 11,159 patients with type 2 diabetes. GI AEs were reported in 41.9% of those receiving sc semaglutide and 39.1% of those receiving oral semaglutide, compared to 22.0% and 24.8%, respectively, in comparator groups, primarily at the initiation of treatment. The incidence of fatal AEs, renal disorders, acute pancreatitis, malignant neoplasms, hypoglycemia, diabetic retinopathy, heart failure, and other cardiovascular events was comparable across all cohorts. Gallstone formation was more common among patients treated with semaglutide, and an increased rate of diabetic retinopathy was observed with sc administration in the SUSTAIN 6 trial. Both formulations resulted in a modest elevation in pulse rate without an increase in arrhythmia risk. Sc semaglutide demonstrated a reduction in major adverse cardiovascular event (MACE) risk, while oral semaglutide was shown to be non-inferior.

## 7. Post-Marketing Evidence and Expansion of Indications

### 7.1. Real-World Effectiveness Studies (Registry and Claims Data)

In their analysis of the US Komodo Health data, Ruseva et al. [65] identified adults with a BMI ≥ 30 kg/m^2^ or those with a BMI between 25 and 29.9 kg/m^2^ accompanied by at least one weight-related comorbidity. Following two years of semaglutide therapy (1.7 or 2.4 mg), results from 179 patients revealed a mean weight reduction of −17.5 kg (−16.2%), while 369 patients experienced a mean decrease in BMI of −6.0 kg/m^2^ (*p* < 0.0001). Notably, the proportions of patients achieving weight reductions of ≥5%, ≥10%, ≥15%, and ≥20% were 90.5%, 69.8%, 46.9%, and 29.6%, respectively. These outcomes demonstrate clinically significant and sustained decreases in both weight and BMI.

### 7.2. STEP Trials and Formal Obesity Indication

The STEP trials played a crucial role in gathering evidence to establish semaglutide’s formal indication for weight management in adults with obesity or overweight, ultimately leading to its approval in the US, Europe, UK, and Canada. These trials collectively showed that a weekly 2.4 mg sc dose, when combined with lifestyle intervention, significantly reduces body weight and improves associated health conditions. Semaglutide is now approved for patients with a BMI of ≥30 kg/m^2^ or a BMI of ≥27 kg/m^2^ with at least one weight-related comorbidity [66].

### 7.3. Ongoing Investigations: Metabolic Dysfunction-Associated Steatohepatitis (MASH), Cardiovascular Prevention, Chronic Kidney Disease

#### 7.3.1. MASH

MASH, formerly known as nonalcoholic steatohepatitis (NASH), is the more rapidly progressive form of MASLD and a leading cause of morbidity and health-related expenditures [67,68,69]. While lifestyle changes are effective in improving liver histology [70] maintaining these changes over the long-term is difficult, justifying pharmacological treatment. Resmetirom, a thyroid hormone receptor beta agonist, has been the first approved drug for MASH treatment, with semaglutide being the second [71,72]. This summarizes the published body of evidence leading to FDA approval of semaglutide for fibrosing MASH. Additional real-world studies are necessary to better define the spectrum of efficacy and safety in clinical practice. Nowadays, there is a lot of evidence showing that semaglutide is therapeutically effective in treating MASLD/MASH (Table 3).

#### 7.3.2. Protection Against Cardiovascular and Renal Disease

The cardiovascular (CV), renal, and metabolic benefits of semaglutide in individuals with and without T2D have recently been reviewed by MacIsaac et al. [81]. These authors found that semaglutide by improving glycemic control, reduces MACE and retards the progression of CKD in T2D. Supporting these notions, the SUSTAIN-6 trial showed a statistically significant 26% reduction in MACE among individuals with T2D at high CV-risk using semaglutide at dosages of 0.5 or 1.0 mg OW. Similarly, the FLOW trial found a statistically significant 24% reduction in major kidney disease events with weekly 1.0 mg semaglutide among subjects living with T2D and CKD. In agreement, Shaman et al. [82] by pooling data from SUSTAIN 6 (Trial to Evaluate Cardiovascular and Other Long-Term Outcomes With Semaglutide in Subjects With Type 2 Diabetes; *n* = 3297) and LEADER (Liraglutide Effect and Action in Diabetes: Evaluation of Cardiovascular Outcome Results; *n* = 9340) assessed the effect of OW semaglutide and OD liraglutide on kidney outcomes in T2D. These authors concluded thatemaglutide/liraglutide offered kidney-protective effects, which appeared more pronounced in patients with preexisting CKD among individuals with T2D.

The SELECT trial [35] highlighted that semaglutide administered sc at a dose of 2.4 mg OW was effective in significantly reducing MACE by 20% and slowing the progression of CKD among non-diabetic overweight or obese individuals with preexisting CV. Additionally, semaglutide improved the symptomatology of heart failure reducing hospital admission among individuals living with obesity regardless of their T2D status [83]. Collectively, these findings underscore semaglutide’s role in improving renal function, CV, and survival outcomes among high-risk patients.

### 7.4. Pharmacovigilance and Long-Term Safety Signals

The safety of OW sc 2.4 mg semaglutide versus placebo, beyond the reduction in MACEs among individuals living with overweight or obesity and exhibiting overt CVD has been evaluated by Kushner et al. [84]. Data has shown that the fraction of individuals experiencing severe AEs (SAEs) was significantly lower with semaglutide than placebo, attributed to a decrease in heart disease. Moreover, semaglutide led to significantly more discontinuations due to AEs than placebo, primarily from GI AEs, while the rates of discontinuations due to serious AEss and suicide/self-injury were not statistically different. Gallbladder disorders occurred significantly more often with semaglutide due to gallstones; conversely, the incidence of cholecystitis was identical. Therefore, this study confirmed that the SELECT study reaffirms the long-term safety profile observed in previous studies, without identifying any novel safety concerns for OW 2.4 mg semaglutide.

Chiappini et al. [85] conducted the first descriptive analysis of the characteristics of Adverse Event Reports (AER) documenting the misuse and abuse potential of semaglutide compared to other GLP-1RAs (e.g., albiglutide, dulaglutide, exenatide, liraglutide, lixisenatide, and tirzepatide) by analyzing the FDA Adverse Events Reporting System (FAERS) pharmacovigilance data set. In the study period (January 2018–December 2022), the most represented reports involved dulaglutide (37.6%), semaglutide (26.1%), and liraglutide (25.0%), totaling 31,439 AERs. Semaglutide exhibited an increased number of reported AERs compared to other GLP-1RAs, with the majority of reports from the US involving female adults. Reports of drug misuse, abuse, and withdrawal were most commonly associated with semaglutide compared to the other selected GLP-1 analogues. Specifically, ‘drug abuse’, ‘drug withdrawal syndrome’, and ‘prescription drug used without a prescription’ were reported more than 3.5 times as frequently, and ‘intentional product use issue’ was reported almost two times as frequently indicating that further studies are essential to determine the extent and type of semaglutide misuse/abuse among the general public and vulnerable sub-groups of individuals.

Finally, the European Medicines Agency (EMA) safety committee Pharmacovigilance Risk Assessment Committee (PRAC) has completed its review of medicines containing semaglutide. This review was prompted by concerns about a potential increased risk of developing non-arteritic anterior ischemic optic neuropathy (NAION), an eye condition that can lead to vision loss. PRAC has determined that NAION is a very rare side effect of semaglutide medicines including Ozempic, Rybelsus and Wegovy [86]. As a result, PRAC has recommended that NAION be included in the product information for semaglutide medicines as a side effect with a frequency of ‘very rare’ (affecting up to 1 in 10,000 people). If patients using semaglutide experience a sudden loss of vision or a rapid deterioration in eyesight, they should promptly contact their doctor. If NAION is confirmed, treatment with semaglutide should be discontinued.

## 8. Translational Insights and Mechanistic Updates

### 8.1. Central Nervous System Pathways Underlying Appetite Suppression

By reviewing the mechanisms through which semaglutide may act centrally and peripherally in regulating appetite and energy homeostasis, Moiz et al. [18] pinpoint that semaglutide exerts an anorexigenic effect by mimicking the hormone GLP-1 in the Central Nervous System. This is achieved through the activation of appetite-suppressing neurons and inhibition of appetite-stimulating neurons in the arcuate nucleus of the hypothalamus, serving as the primary mechanism of action, as well as in other brain areas. Furthermore, semaglutide also contributes to appetite control by impacting dopamine reward signaling, gastric emptying, and influencing other cerebral pathways. Additionally, semaglutide may act synergistically with the hormone leptin to reduce food intake and increase energy expenditure.

### 8.2. β-Cell Preservation and Insulin Secretory Dynamics

A monocentric double-blind, placebo-controlled, parallel-group trial was conducted by Kapitza et al. [87]. These authors enrolled 75 adults with T2D who were being treated with diet and exercise and/or metformin monotherapy. The participants were randomly assigned to OW s.c. semaglutide 1.0 mg (*n* = 37) or a placebo (*n* = 38). The data showed significant improvement in β-cell function and glycemic control, as assessed by fasting, postprandial, and overall (AUC_0–24h_) glucose and glucagon responses, as well as insulin secretion rate, among those with T2D who received semaglutide.

### 8.3. Anti-Inflammatory and Cardio-Renal Protective Mechanisms

Semaglutide exerts anti-inflammatory and cardio-renal protective mechanisms through direct and indirect effects. It down-regulates systemic inflammation by modulating immune responses in adipose tissue and inhibiting pro-inflammatory pathways such as interleukin-6, tumor necrosis factor-α, and S100A8/A9 [88].

The anti-inflammatory and anti-atherogenic properties of semaglutide, along with reductions in blood pressure, body weight, and improvements in lipid profile, contribute to cardiovascular protection [89]. Direct renal protection is achieved through decreased inflammation, fibrosis, and albuminuria, while renal protection is achieved through improved glycemic homeostasis, blood pressure, and body weight [90]. Together, these mechanisms significantly reduce the risk of CKD progression, kidney failure, or cardiovascular death in patients with T2D and CKD [35].

## 9. Future Directions

### 9.1. Oral Formulations and Next-Generation Analogues

Despite the extensive SUSTAIN and STEP programs, several knowledge gaps remain. First, most trials have only followed participants for up to 2 years. Longer surveillance is necessary to understand the durability of glycemic control, cardiovascular protection and the extent of weight regain after drug discontinuation, as seen in extension studies. Second, the mechanisms behind inter-individual variability in efficacy and GI tolerability are not well understood. Multi-omics profiling (genomics, metabolomics, microbiomics) could help identify predictive biomarkers to guide dose selection and reduce dropouts. Third, there is still limited pediatric data, especially regarding long-term effects on bone accrual, pubertal development and neuro-cognitive endpoints in children and adolescents. Lastly, while semaglutide is being explored beyond diabetes and obesity, phase II and III programs must confirm benefits seen in surrogate markers translate to actual clinical outcomes.

Chemical innovation surrounding the GLP-1 pharmacophore is progressing rapidly. Oral semaglutide tablets are already on the market, and next-generation analogues with dual or triple receptor activity (e.g., GLP-1/GIP or GLP-1/glucagon co-agonists) show promise for greater weight reduction [91]. Artificial intelligence-driven approaches that integrate deep learning-based protein design with functional screening for isolation of long-acting GLP-1 RA are on the way to help in designing novel compound with improved stability and efficacy [92]. Combination therapies are also being explored, such as a phase II study combining semaglutide with the FXR agonist cilofexor and the ACC inhibitor firsocostat, which showed greater improvements in hepatic steatosis and metabolic biomarkers compared to semaglutide alone. In addition, digital therapeutics, mobile applications that offer dietary coaching, physical-activity tracking and adherence prompts, could help reinforce lifestyle changes and prevent post-withdrawal weight regain. Lastly, the production of biosimilars after patent exclusivity ends, or the use of compulsory-licensing statutes by governments, may increase access and control costs in high-prevalence settings.

### 9.2. Combination Therapies

An emerging strategy to amplify the clinical impact of semaglutide is to administer it alongside peptide partners that engage complementary metabolic pathways [26,91]. The leading example is its co-development with cagrilintide (formerly AM833), a long-acting analogue of amylin, the hormone co-secreted with insulin that slows gastric emptying, suppresses glucagon after meals and enhances meal-related satiety. Whereas semaglutide acts predominantly through GLP-1 receptors to boost glucose-dependent insulin secretion and centrally dampen appetite, cagrilintide stimulates amylin receptors located in distinct hypothalamic and brain-stem nuclei [93]. Pre-clinical studies have shown that the two signals converge additively, producing larger body-weight reductions than either peptide alone and without reciprocal attenuation of glucoregulatory effects.

This concept has already advanced to the clinic. In a multinational phase-II trial, adults with obesity received OW semaglutide 2.4 mg combined with escalating doses of cagrilintide. After 32 weeks, mean weight loss in the highest-dose cohort reached approximately 17–18% of baseline, outstripping the ≈14% loss observed with semaglutide monotherapy; glycemic, blood-pressure and lipid endpoints improved in parallel. The GI profile (chiefly nausea and vomiting) was slightly more pronounced in the combination group, but most events were transient and of mild-to-moderate severity, leading to discontinuation rates comparable with the single-agent arm [26]. These data have triggered a global phase-III program (REDEFINE) that will test long-term cardiovascular and renal outcomes as well as durability of weight control [94].

Pharmacologically, the peptide pair is well-matched: both molecules have been engineered for OW sc injection via albumin-binding fatty-acid acylation, allowing co-formulation without complex titration schedules. No relevant pharmacokinetic interaction has been detected to date. Nevertheless, careful dose-escalation remains essential because the overlapping satiety signals can potentiate early GI. Of note, modelling work suggests that combined GLP-1 and amylin receptor agonism may attenuate the post-treatment weight regain sometimes observed after semaglutide withdrawal, an effect already hinted at in extension cohorts.

The cagrilintide partnership is not the only avenue for combination therapy [39]. A phase II proof-of-concept study in non-alcoholic steatohepatitis combined semaglutide with the FXR agonist cilofexor, and the acetyl-CoA carboxylase (ACC) inhibitor firsocostat [95]. The triple regimen produced larger reductions in hepatic fat content and broader metabolic benefits than semaglutide alone while maintaining acceptable tolerability. Parallel investigations are pairing semaglutide with SGLT2 inhibitors, GIP co-agonists and centrally acting appetite suppressants, all aiming to transcend the ceiling of efficacy achievable through single-pathway modulation.

From a regulatory perspective, the use of two peptides that already possess extensive safety dossiers may permit streamlined approval pathways, provided that post-marketing pharmacovigilance confirms the absence of novel safety liabilities. Economically, the dual-peptide approach will face scrutiny because of its higher acquisition cost, yet modeling analyses suggest that deeper and more durable weight loss could shorten time-to-goal and lower downstream cardiovascular and renal expenditures, partially offsetting upfront spending.

Ultimately, biomarker-guided precision medicine, leveraging baseline amylin levels, eating-behavior phenotypes or genetic polymorphisms in amylin-receptor signaling, may identify the sub-populations most likely to derive incremental benefit, thereby maximizing the therapeutic and economic value of GLP-1/amylin co-therapy. As such, the semaglutide-cagrilintide paradigm exemplifies how layering complementary hormonal cues can push pharmacological weight loss toward the territory historically reserved for bariatric surgery, while preserving the established cardiometabolic advantages of GLP-1 receptor agonism. This blueprint is rapidly being emulated by other combination strategies that seek to deliver multidimensional, disease-modifying benefits for complex metabolic disorders.

### 9.3. Precision Medicine: Biomarkers to Predict Responders

A striking feature of the SUSTAIN and STEP programs is the broad dispersion of individual outcomes: some participants lose more than 20% of their initial body weight, whereas others achieve only modest reductions despite identical dosing and adherence. Understanding this heterogeneity is pivotal for maximizing therapeutic benefit and for containing the substantial drug-acquisition costs that currently limit access.

The search for predictive biomarkers begins with pharmacogenetics. Single-nucleotide polymorphisms in GLP-1R or in genes governing peptide trafficking (e.g., DPP4, albumin) could modulate receptor signaling, degradation kinetics or tissue distribution, thereby influencing both efficacy and GI tolerability. Early sequencing studies have already linked certain GLP-1R variants to differential HbA1c responses with older incretin analogues; prospective genotyping in forthcoming semaglutide trials should clarify clinical relevance. Beyond fixed DNA markers, dynamic “omics” layers offer additional discriminatory power. Baseline metabolomic signatures that reflect hepatic de novo lipogenesis, branched-chain amino-acid catabolism, or bile-acid pools may stratify patients by their propensity to mobilize adipose stores under GLP-1 agonism. Likewise, gut-microbiome profiles enriched in *Prevotella* or *Bacteroides* species—taxa implicated in short-chain fatty-acid production and energy harvest—might predict the degree of weight loss achieved when appetite is pharmacologically suppressed.

Clinical phenotyping remains equally informative. Patients with pronounced visceral adiposity, high baseline fasting insulin, or rapid gastric emptying have shown larger weight-loss increments and fewer hypoglycemic events in post hoc analyses. Continuous glucose-monitoring data further reveal that responders display an early, sharp attenuation of post-prandial excursions within the first two weeks, whereas non-responders exhibit a flatter trajectory. Incorporating those early digital biomarkers into adaptive algorithms could allow dose intensification or timely switch to alternative therapies.

Safety prediction is another frontier. Polymorphisms in serotonin-receptor genes, variants in the calcitonin pathway, or pre-existing dysbiosis characterized by methane-producing archaea may forecast susceptibility to nausea, gall-bladder events, or constipation, respectively. Pre-emptive identification of such risk factors would enable slower titration schedules, prophylactic anti-emetics, or selection of alternative weight-management strategies.

Implementation science will ultimately determine the clinical utility of these candidate markers. Pragmatic trials that embed genotyping, metabolomics, and microbiome sequencing into routine care can generate real-world evidence on cost-effectiveness. Machine-learning models fed with multi-modal data—demographics, genetics, laboratory values, CGM streams, wearable-derived activity metrics—can then produce individualized response probabilities delivered seamlessly to electronic health-record systems. Such precision triage is especially pertinent given recent estimates that 4.5 million U.S. adults meet eligibility criteria for semaglutide therapy: even modest improvements in response rates or discontinuation avoidance would translate into substantial absolute gains in population health and budgetary sustainability.

In short, biomarker-guided precision medicine offers a pragmatic path to amplify the impact of semaglutide while safeguarding health-system resources. By identifying likely responders, forecasting adverse-event risk, and informing dynamic dose adjustments, such an approach can convert a “one-size-fits-all” injectable into a tailored intervention fully aligned with the principles of value-based metabolic care.

### 9.4. The Role of Personalized Medicine

The precise use of semaglutide is likely to improve both effectiveness and cost-efficiency. Algorithms that consider baseline BMI category, visceral-fat distribution, renal function, gut-hormone profiles and behavioral traits could help predict responders versus non-responders. This information could also guide decisions on whether monotherapy, combination therapy or bariatric surgery is the most appropriate course of action. Continuous glucose monitoring and smart-pen data streams can be used to adjust doses in real-time using machine learning models and shape diagnostics [96]. Similarly, analyzing patient reviews of semaglutide, with the goal of better understanding its real-world effectiveness and safety for weight management might help with surveillance and improve individualized regimens [97]. This approach can help maintain glycemic targets while minimizing GI AEs. Furthermore, pharmacogenetic screening for variants that affect peptide clearance or GLP-1 receptor signaling may eventually support genotype-directed titration schedules. With up to 4.5 million U.S. adults meeting eligibility criteria for semaglutide therapy, it will be crucial to deploy this treatment in a stratified manner to maximize its public health impact within limited budgets.

## 10. Conclusions

The development of semaglutide from an engineered GLP-1 analogue in the laboratory to a globally approved, first-line therapy for diabetes and obesity illustrates the power of modern translational pharmacology. By combining rational peptide design, strategic amino-acid substitutions and albumin-binding acylation, with systematic, hypothesis-driven experimentation, investigators achieved a pharmacokinetic profile (approximately 160 h half-life) that made OW dosing clinically feasible without compromising receptor potency or safety. Pre-clinical models consistently showed durable glycemic and weight-loss effects, paving the way for expedited entry into human studies.

Phase I and II trials subsequently confirmed the predictable pharmacokinetics, dose-proportional efficacy, and manageable tolerability profile of semaglutide, allowing clear selection of therapeutic doses (0.5 mg and 1 mg s.c. weekly). The SUSTAIN Phase III program then provided unequivocal evidence of superior HbA1c lowering, meaningful weight reduction, and, critically, cardiovascular risk mitigation, culminating in a 26% relative risk reduction in major adverse cardiovascular events in SUSTAIN-6. These findings established semaglutide not merely as a glucose-lowering drug but as a disease-modifying agent in metabolic medicine. The subsequent STEP trials extended its utility to chronic weight management, with ≥15% body-weight loss in a sizable proportion of patients, thus addressing an adjacent yet intertwined public-health challenge.

Beyond the initial approvals (FDA 2017, EMA 2018), real-world evidence confirms the clinical benefits of semaglutide, while ongoing post marketing has thus far revealed no unexpected safety signals. Current investigations span MASH, CKD, and heart-failure prevention, underscoring the pleiotropic potential of this drug. The parallel development of oral semaglutide and combination regimens, such as the shared use of semaglutide and cagrilintide, points to an even broader achievable therapeutic impact.

In accordance with clinical guidelines, most trials administering GLP1-RAs have also utilized adjunct behavioral therapy to enhance treatment outcomes. However, information on these supplementary therapies is often limited. Behavioral interventions can also increase the overall costs, highlighting the importance of future research to determine the most effective and cost-efficient support protocols.

We also believe that time restricted semaglutide treatment may lead to weight gain recurrences upon drug withdrawal unless significant lifestyle changes are made during the treatment period. Strict eligibility criteria may limit access to semaglutide, potentially causing disparities in prescriptions in private medical practices. These policies and access limitations could impact the real-world effectiveness and sustainability of treatment benefits.

Additionally, semaglutide, like other anti-obesity medications, has the potential to distract physicians, the public, and policymakers from broader strategies aimed at preventing and addressing the global obesity epidemic.

Several lessons emerge for future drug discovery efforts:

- Rational molecular tailoring can overcome intrinsic limitations of endogenous peptides.
- Early alignment of pre-clinical endpoints with clinically meaningful outcomes accelerates translation.
- Cardiovascular outcome trials, once considered ancillary, can strategically reposition metabolic drugs as cardioprotective agents.
- Long-term safety surveillance remains essential, especially for agents that modulate appetite and body weight.

In sum, semaglutide exemplifies a successful bench-to-bedside journey, reshaping therapeutic strategies for both T2Ds and obesity. Its development pathway offers a replicable blueprint for tackling other complex, multifactorial diseases, anchored in meticulous molecular engineering, rigorous pre-clinical validation, outcome-oriented clinical trials, and sustained post-marketing attention.

## Figures and Tables

**Table 1:** Commercial names, indications and administration of GLP-1RAs (modified from [23]).

Drug	Brand Name	FDA-Approved Indication	Modality of Administration
Exenatide	Byetta	T2D	Sc TD
	Bydureon	T2D	Sc OW
Liraglutide	Victoza	T2D	Sc OW
	Saxenda	Obesity	
Dulaglutide	Trulicity	T2D	Sc OW
Lixisenatide	Adlixyn	T2D	Sc OD
Semaglutide	Ozempic	T2D	Sc OW
	Ribelsus	T2D	Oral, OD
	Wegovy	Obesity; MASH	Sc OW
Tirzepatide	Mounjaro	T2D	Sc OW
	Zepbound	Obesity	

Abbreviations: MASH, metabolic dysfunction-associated steatohepatitis; OD, once daily; OW, once weekly; Sc, subcutaneous; T2D, type 2 diabetes; TD, twice daily.

**Table 2:** Major milestones in the development of semaglutide.

Year	Milestone Category	Key Study/Regulatory Event	Principal Outcome or Significance
2008–2014	Target identification & lead optimization	GLP-1 receptor biology clarified; iterative peptide engineering (Aib8 substitution, C18 di-acid side-chain, Lys26 conjugation)	Established DPP-4 resistance and albumin binding, enabling ~160 h half-life and once-weekly dosing.
2015	Molecule discovery	First disclosure of semaglutide structure [32]	Publication of rational design that preserved full GLP-1 activity with markedly prolonged pharmacokinetics
2015	IND-enabling package	GLP-compliant pharmacology & toxicology studies in rodents/primates	Demonstrated glucose lowering, weight loss, β-cell protection and acceptable safety margin, supporting first-in-human trials
2015–2016	Phase I (FIH)	Single- and multiple-ascending-dose trials [33]	Confirmed ~1 week half-life, dose-proportional exposure, and good tolerability; supported once-weekly sc and once-daily oral regimens
2016	Phase II dose-finding	12-week RCT in T2D [34]	Identified optimal 0.5 mg and 1.0 mg sc once-weekly doses with robust HbA1c and weight reductions
2017	First regulatory approval	FDA approval of sc semaglutide (Ozempic) for T2D	Marked entry into clinical practice for glycaemic control
2016–2018	Pivotal glycaemic efficacy (SUSTAIN 1-5, 7)	>12,000 patients with T2D	Superior HbA1c lowering (up to −1.8%) and significant weight loss compared to comparators
2016 (reported)/2018	Cardiovascular outcome	SUSTAIN-6 CVOT	26% relative risk reduction in MACE, confirming CV safety and benefit
2019	First oral GLP-1RA	PIONEER program resulting in FDA approval of oral semaglutide (Rybelsus) for T2D	Demonstrated efficacy of peptide in tablet form; expanded patient options
2021	Weight-management efficacy	STEP 1-4 trials; FDA approval of 2.4 mg sc semaglutide (Wegovy)	≈15% mean body-weight reduction; first GLP-1RA approved specifically for obesity
2023	Cardiovascular benefit in non-diabetic obesity	SELECT trial [35]	20% reduction in MACE in people with obesity and CV disease but without diabetes
2024	Renal outcomes	FLOW trial [36]	24% reduction
2025	Liver indication & expanded safety monitoring	Phase III MASH trial [37] resulting in FDA approval for fibrosing MASH; EMA PRAC adds NAION as “very rare” side effect	Positions semaglutide as second approved drug for MASH; continued vigilance for ocular safety

Abbreviations: Aib, 2-Aminoisobutyric acid; CKD, chronic kidney disease; CV, cardiovascular; CVOT, cardiovascular outcomes trial; DPP-4, dipeptidyl-peptidase-4; EMA PRAC, European Medicines Agency Pharmacovigilance Risk Assessment Committee; FDA, U.S. Food and Drug Administration; FIH, first-in-human; GLP-1, glucagon-like peptide-1; IND, Investigational New Drug (application); MACE, major adverse cardiovascular events; MASH, metabolic dysfunction-associated steatohepatitis; NAION, non-arteritic anterior ischemic optic neuropathy; RCT, randomized controlled trial; sc, subcutaneous; T2D, type 2 diabetes.

**Table 3:** Evidence supporting the effectiveness of semaglutide in MASLD/MASH.

Author, Year [Ref]	Method	Findings	Conclusion
Katrevula et al., 2025 [73]	An open-label, RCT recruiting 116 adults with a BMI ≥ 30 or ≥27 with comorbidities (pre-diabetes, hypertension, dyslipidemia, obstructive sleep apnea or cardiovascular disease) randomized into two groups (*n* = 58 per group), both receiving counselling on hypocaloric diet and increased physical activity. Group 1 also received 3 to 14 mg/day of oral semaglutide.	At 28 weeks, semaglutide administration induced a higher mean percentage weight reduction compared to controls. There were significantly higher improvements in BMI, WC, HbA1c, fasting insulin, CRP, and total fat mass decrease, as well as improved ALT and GPT levels, reductions in the APRI score, LFC and liver stiffness.	In non-diabetic adults living with overweight or obesity, oral semaglutide combined with dietary and lifestyle modifications compared to lifestyle modifications alone led to statistically significant and clinically meaningful loss of body weight and metabolic improvements.
Nitze et al., 2025 [74]	Retrospective study exploring NITs for assessing the response to semaglutide treatment in 268 patients with MASH randomised in a phase 2b trial who remained on treatment throughout the trial and had liver biopsy and NIT results at baseline and week 72.	Treatment with Semaglutide, compared to placebo, was associated with significant reductions in all NIT scores. More patients exhibited MASH improvement and fewer had fibrosis progression.	NITs may be used to evaluate treatment responses in MASH patients submitted to semaglutide treatment.
John et al., 2025 [75]	A target trial was emulated using the electronic health records of 8040 US Veterans with positive AUDIT-C comparing new initiators of GLP-1 RA between 3 January 2017 and 30 September 2024, with 8040 non-initiator controls, with follow-up until outcomes (decompensation, hepatocellular carcinoma, liver-related death, and all-cause mortality) or study end.	GLP-1 RA use was associated with a lower risk of composite liver-related outcomes and death. A 1 mg/wk increase in semaglutide dose was associated with a reduced risk of composite liver-related outcomes, death, and lower odds of positive AUDIT-C during follow-up.	This OTTES shows that GLP-1 RA use protects from MALO adverse liver outcomes, death, and harmful alcohol use
Sanyal et al., 2025 [37]	Phase 3, multicenter, double-blind RCT assigning 1197 patients with biopsy-proven MASH and fibrosis stage 2 or 3 to receive OW sc 2.4 mg semaglutide or placebo for 240 weeks.	The proportions of SH resolution without worsening of fibrosis, reduced liver fibrosis without SH worsening, combined SH resolution and liver fibrosis reduction in liver fibrosis and mean decrease in body weight were all more elevated with semaglutide than with placebo (*p* < 0.001 for all).	Among MASH individuals with moderate-advanced liver fibrosis, OW 2.4 mg semaglutide improved liver histology outcomes and decreased body weight.
Golub et al., 2025 [76]	114 individuals were enrolled (59 in the semaglutide group and 55 in the placebo group) and followed for 12 months. Liver fat attenuation was quantified with non-contrast cardiac CT scanning at baseline and after 12 months.	In multivariable analysis adjusted for demographic and metabolic variables, smoking and baseline liver attenuation average liver attenuation measures improved by 4.4 HU in the semaglutide group vs. placebo (*p* = 0.002).	Among individuals with T2D semaglutide administration resulted in a significant reduction in SLD vs. placebo.
Ratziu et al., 2024 [77]	A post hoc analysis of 251 individuals with biopsy-proven NASH and fibrosis stage F1-F3 from a 72-week RCT of OD sc semaglutide (0.1, 0.2, or 0.4 mg).	Pathologist and ML-derived assessments detected a significantly higher proportion of individuals achieving NASH resolution without worsening of fibrosis with semaglutide 0.4 mg versus placebo (pathologists *p* < 0.0001; ML *p* = 0.0015). ML continuous scores detected significant treatment-associated quantitative reduction in fibrosis with semaglutide 0.4 mg versus placebo (*p* = 0.0099).	ML categorical assessments overlapped with pathologists’ results of histological improvement for steatosis and disease activity among those assigned to semaglutide. ML-based continuous scores demonstrated an antifibrotic effect not identified with conventional histopathology
Loomba et al., 2023 [78]	A double-blind, placebo-controlled multi-centre phase 2 trial enrolling 71 patients with biopsy-proven NASH- cirrhosis and BMI of 27 kg/m^2^.	After 48 weeks, there was no statistically significant difference between the two groups in the proportion of patients with an improvement in liver fibrosis of one stage or more without worsening of NASH nor between groups in the proportion of patients who achieved NASH resolution. Similar proportions of patients in each group reported adverse events and serious adverse events. Hepatic and renal function remained stable. There were no decompensating events or deaths.	In patients with compensated NASH-cirrhosis, semaglutide compared to placebo did not significantly improve fibrosis or achieve NASH resolution. No new safety concerns were raised.
Fint et al., 2021 [79]	An RCT of 67 subjects with LSM 2.50–4.63 kPa by MRE and liver steatosis ≥10% by MRI-PDFF randomised to OD sc semaglutide 0.4 mg (*n* = 34) or placebo (*n* = 33).	Reductions in liver steatosis were significantly greater with semaglutide (*p* < 0.0001) and more subjects achieved a ≥30% reduction in liver fat content with semaglutide at weeks 24, 48 and 72, (all *p* < 0.001). Decreases in liver enzymes, body weight and HbA1c were also observed with semaglutide. However, changes from baseline in LSM were not significantly different between semaglutide and placebo at week 24, 48, and 72.	Among NAFLD subjects, semaglutide administration was not associated with reduced LSM vs. placebo. However, compared to placebo semaglutide significantly reduced liver steatosis and improved liver enzymes and metabolic parameters.
Newsome et al., 2021 [80]	72-week, double-blind phase 2 trial involving a total of 320 individuals with biopsy-proven NASH and liver fibrosis of stage F1, F2, or F3 randomized to receive semaglutide at a dose of 0.1 mg (80 patients), 0.2 mg (78 patients), or 0.4 mg (82 patients) or placebo (80 patients).	The proportion of individuals who achieved NASH resolution with no worsening of fibrosis was higher for semaglutide 0.4 mg vs. placebo (*p* < 0.001). Improved fibrosis stage occurred in statistically similar proportions of the patients in the 0.4 mg group compared to the placebo group. The mean percent weight loss was 13% in the 0.4-mg group and 1% in the placebo group. The incidence of GI side effects was higher in the 0.4-mg group vs. placebo. Cancers were diagnosed in 3 patients who received semaglutide vs. no patients assigned to placebo. Overall, benign, malignant, or unspecified neoplasms were identified in 15% of the subjects assigned to semaglutide vs. 8% of the placebo group, without any organ-specific pattern of occurrence.	Semaglutide resulted in a significantly higher proportion of NASH resolution compared to a placebo, with no significant difference between groups in the proportion of those achieving improved fibrosis stages.

List of abbreviations used: ALT—alanine transaminase; AST—aspartate transaminase; APRI—aspartate aminotransferase to platelet ratio index; AUDIT-C—alcohol use disorders-concise score; BMI—body mass index; CRP—C-reactive protein; CT—computed tomography; GLP-1 RA—glucagon-like peptide 1 receptor agonist; HbA1c—glycated hemoglobin; HU—Hounsfield Units; LFC—liver fat content; kPa—kilopascal; LSM—liver stiffness measurement; MALO adverse liver outcomes; MASH—metabolic dysfunction-associated steatohepatitis; ML—machine-learning; MRE—magnetic resonance elastography; MRI-PDFF—magnetic resonance imaging-proton density fat fraction; NAFLD—nonalcoholic fatty liver disease; NASH—nonalcoholic steatohepatitis; NITs—Non-invasive tests; OTTES—observational target trial emulation study; OD—once daily; OW—once weekly; RCT—randomized controlled trial; SH—steatohepatitis; SLD—steatotic liver disease; STOP—semaglutide treatment effect on coronary atherosclerosis progression.

## Data Availability

No new data were generated in this study.

## Abbreviations

The following abbreviations are used in this manuscript:

ACC	acetyl-CoA carboxylase
AER	adverse event report
AEs	adverse events
ALT	alanine transaminase
AST	aspartate transaminase
APRI	aspartate aminotransferase to platelet ratio index
AUDIT-C	alcohol use disorders-concise score
BMI	body mass index
CKD	chronic kidney disease
CRP	c-reactive protein
CT	computed tomography
CV	cardiovascular
DPP-4	dipeptidyl-peptidase-4
EMA	European Medical Agency
FAERS	FDA Adverse Events Reporting System
FDA	Food and drug administration
FXR	farnesoid X receptor
GLP-1 RA	glucagon-like peptide 1 receptor agonist
GI	gastrointestinal
GIP	gastrointestinal peptide
HbA1c	glycated hemoglobin
HU	hounsfield unit
kPa	kilopascal
LFC	liver fat content
LSM	liver stiffness measurement
MALO	major adverse liver outcome
MASH	metabolic dysfunction-associated steatohepatitis
ML	machine learning
MRE	magnetic resonance elastography
MRI-PDFF	magnetic resonance imaging-proton density fat fraction
NAFLD	nonalcoholic fatty liver disease
NAION	non-arteritic anterior ischemic optic neuropathy
NASH	nonalcoholic steatohepatitis
NITs	Non-invasive tests
OTTES	observational target trial emulation study
OD	once daily
OW	once weekly
PRAC	Pharmacovigilance Risk Assessment Committee
RCT	randomized controlled trial
sc	subcutaneous
SELECT	semaglutide effects on cardiovascular outcomes in people with overweight or obesity
SH	steatohepatitis
SLD	steatotic liver disease
STOP	semaglutide treatment effect on coronary atherosclerosis progression
SUSTAIN	semaglutide unabated sustainability in treatment of type 2 diabetes
T2D	type 2 diabetes
